# Intrauterine Herpes Simplex Virus Infection: Insights Into a Silent Threat

**DOI:** 10.7759/cureus.78173

**Published:** 2025-01-29

**Authors:** Íris Oliveira, Andreia Fernandes, Mafalda J Pereira, Joana Capela, Claúdia Calado

**Affiliations:** 1 Pediatrics, Unidade Local de Saúde do Algarve - Hospital de Faro, Faro, PRT; 2 Pediatric and Neonatal Intensive Care Unit, Unidade Local de Saúde do Algarve - Hospital de Faro, Faro, PRT

**Keywords:** central nervous system malformations, congenital transmission, cutaneous manifestations, herpes simplex virus, intrauterine hsv infection

## Abstract

We report a rare case of congenital herpes simplex virus type 2 (HSV-2) infection in a neonate born to a mother with no clinical evidence of genital HSV infection either before or during pregnancy. Routine prenatal ultrasound assessments in the first two trimesters were unremarkable, and infectious disease screening results were negative. The pregnancy, however, was complicated by premature rupture of membranes (PROM) at 24 weeks of gestation. An ultrasound at that time revealed severe fetal malformations, including brain abnormalities and organomegaly. At 31 weeks, an emergency cesarean section was performed, delivering a female baby with profound physical abnormalities, including extensive skin erosions and severe neurological impairment. On the fourth day of life, grouped vesicles emerged, leading to the initiation of intravenous acyclovir therapy. Polymerase chain reaction testing of skin swabs confirmed HSV-2 infection. Despite antiviral treatment, the neonate’s condition rapidly deteriorated, culminating in death on the fifth day of life.

This case highlights the diagnostic challenges of intrauterine HSV infection in the absence of maternal symptoms. It emphasizes the importance of considering HSV infection in the differential diagnosis when PROM is accompanied by significant fetal malformations.

## Introduction

Neonatal herpes simplex virus (HSV) infection is a rare condition associated with significant morbidity and mortality [1]. The majority of cases (85%) are acquired in the peripartum period, while a smaller proportion occurs postnatally (10%) or in utero (5%) [2]. Intrauterine HSV infection is predominantly attributed to maternal primary infection, resulting in viremia and transplacental viral transmission [3,4].

Congenital HSV-1 and HSV-2 infections can occur at any stage of pregnancy. Clinical manifestations may be evident at birth or arise within the first 48 hours of life. The classic triad of cutaneous, neurologic, and ophthalmologic findings is only found simultaneously in one-third of affected neonates [5,6]. Cutaneous manifestations are variable, ranging from new vesicles, ulcerations, or rashes to older lesions in various stages of healing (aplasia, crusted papules, and/or hypo/hyperpigmentation) [4]. Ophthalmologic findings may include chorioretinitis, microphthalmia, cataracts, keratoconjunctivitis, and optic nerve atrophy. Neurologic involvement, characterized by intracranial calcifications, encephalopathy, microcephaly, and hydranencephaly, is associated with long-term impairment and high mortality [5].

The risk of neonatal HSV transmission is elevated in cases involving the HSV-1 serotype, primary HSV genital lesions (compared with secondary genital and recurrent lesions), absence of HSV antibodies, vaginal delivery, prolonged rupture of membranes, and compromised fetal skin barriers due to instrumentation at birth [2,5].

## Case presentation

We present the case of a female neonate born to a 42-year-old gravida 4 para 3 woman with an unremarkable medical history. Prenatal ultrasounds in the first and second trimesters were normal, and maternal serological screening demonstrated immunity to rubella and toxoplasmosis and were negative for hepatitis B and C, human immunodeficiency virus, and syphilis. The mother had no previous history of sexually transmitted infections or clinical signs of genital HSV infection during or before pregnancy.

Pregnancy was complicated by premature rupture of membranes (PROM) at 24 weeks of gestation. An ultrasound performed on the day of PROM revealed severe fetal anomalies, including severe cerebral malformations, anasarca, pleural and pericardial effusion, cardiomegaly, and hepatomegaly with intrahepatic calcifications. Infectious testing for parvovirus, cytomegalovirus (CMV), and Zika virus were negative. Amniocentesis revealed a normal microarray analysis with no evidence of aneuploidy. Despite the poor prognosis associated with these findings, the parents opted to continue the pregnancy.

**Figure 1:**
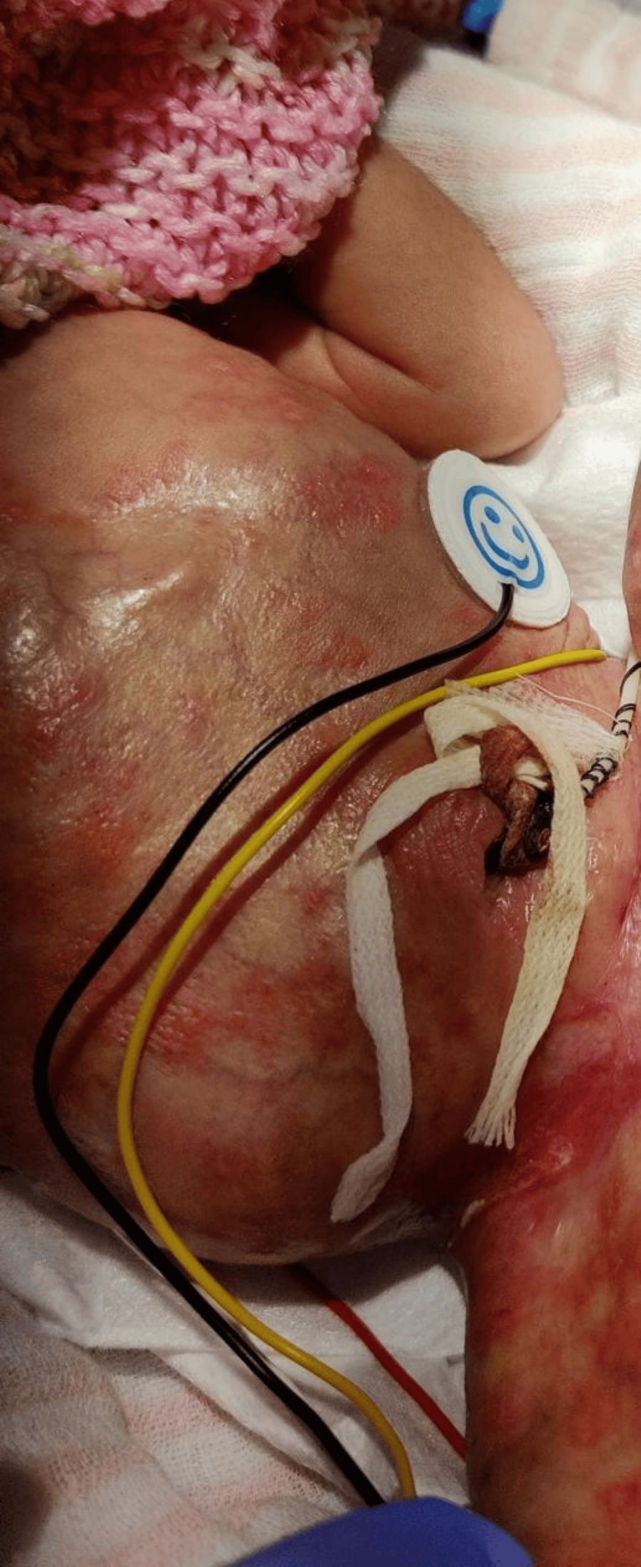
Diffuse skin erosions Diffuse skin erosions on the right lower abdomen and the right lower limb

The baby was born via cesarian section at 31 weeks and 3 days of gestation, weighing 1130 g, with a head circumference of 23 cm (<3rd percentile). Apgar scores were 8, 10, and 10 at 1, 5, and 10 minutes, respectively. Physical examination revealed diffuse skin erosions in various stages of evolution on the right lower abdomen, right forearm, and lower limbs (Figure 1).

**Figure 2:**
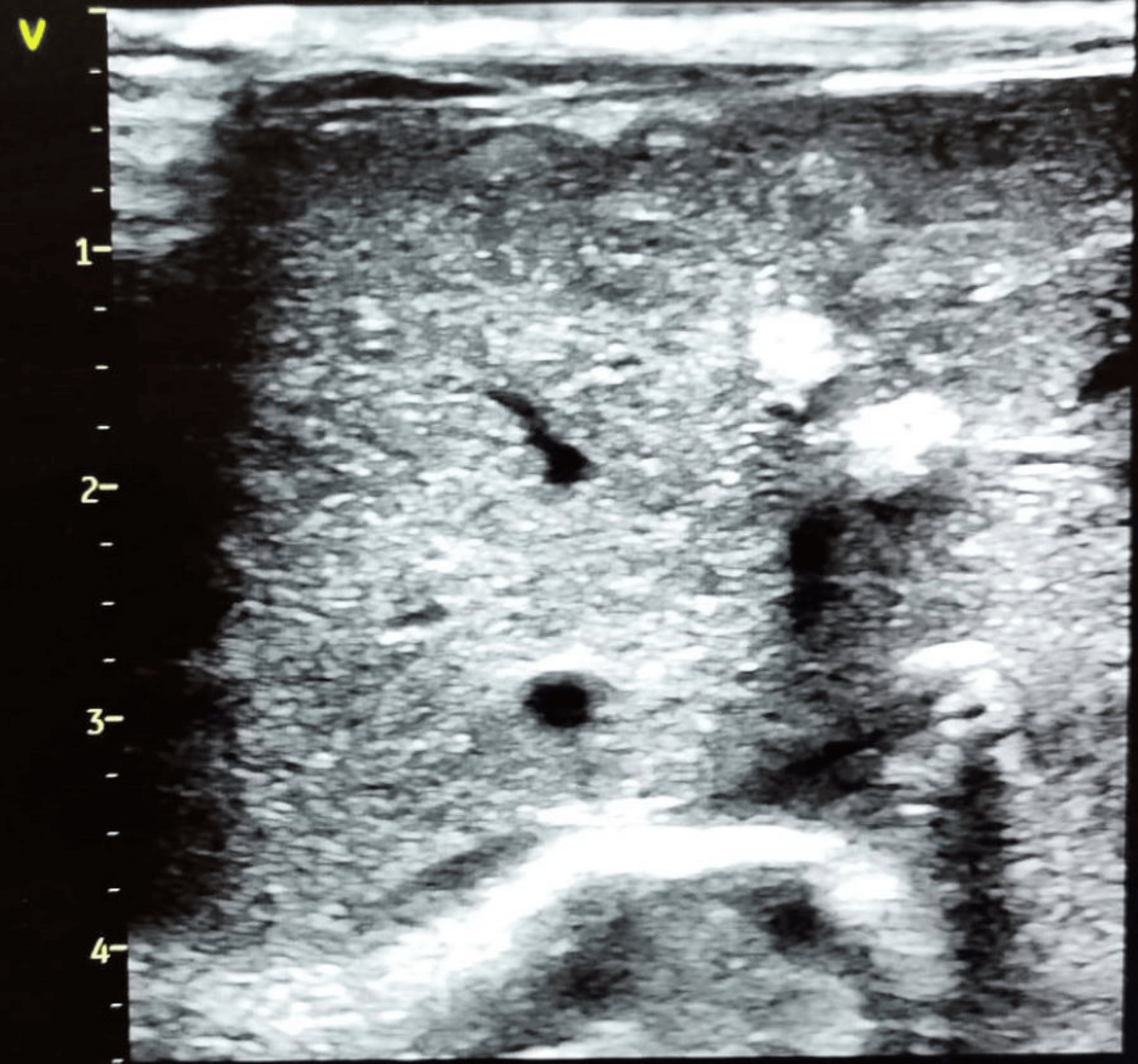
Abdominal ultrasound Abdominal ultrasound showing multiple hyperechoic images consistent with calcifications

**Figure 3:**
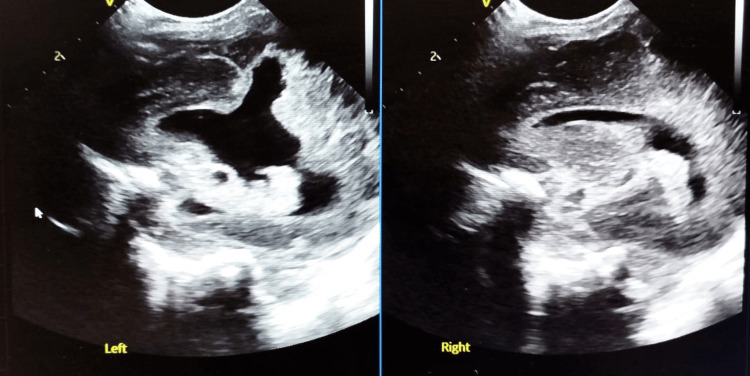
Cranial ultrasound sagittal planes Cranial ultrasound sagittal planes showing CNS malformations, with asymmetric cerebral hemispheres

Umbilical venous catheter was inserted. Initial laboratory studies showed a white blood cell count of 12,400/uL (43% neutrophils, 62% lymphocytes) and a negative C-reactive protein (CRP). Empirical antibiotic therapy with ampicillin and gentamicin was initiated following blood culture collection. Polymerase chain reaction (PCR) testing for CMV and parvovirus was negative. An abdominal ultrasound demonstrated multiple hepatic, suprarenal, and kidney calcifications (Figure 2), while a cranial ultrasound confirmed severe CNS malformations (Figure 3). No ophthalmological examination was performed.

**Figure 4:**
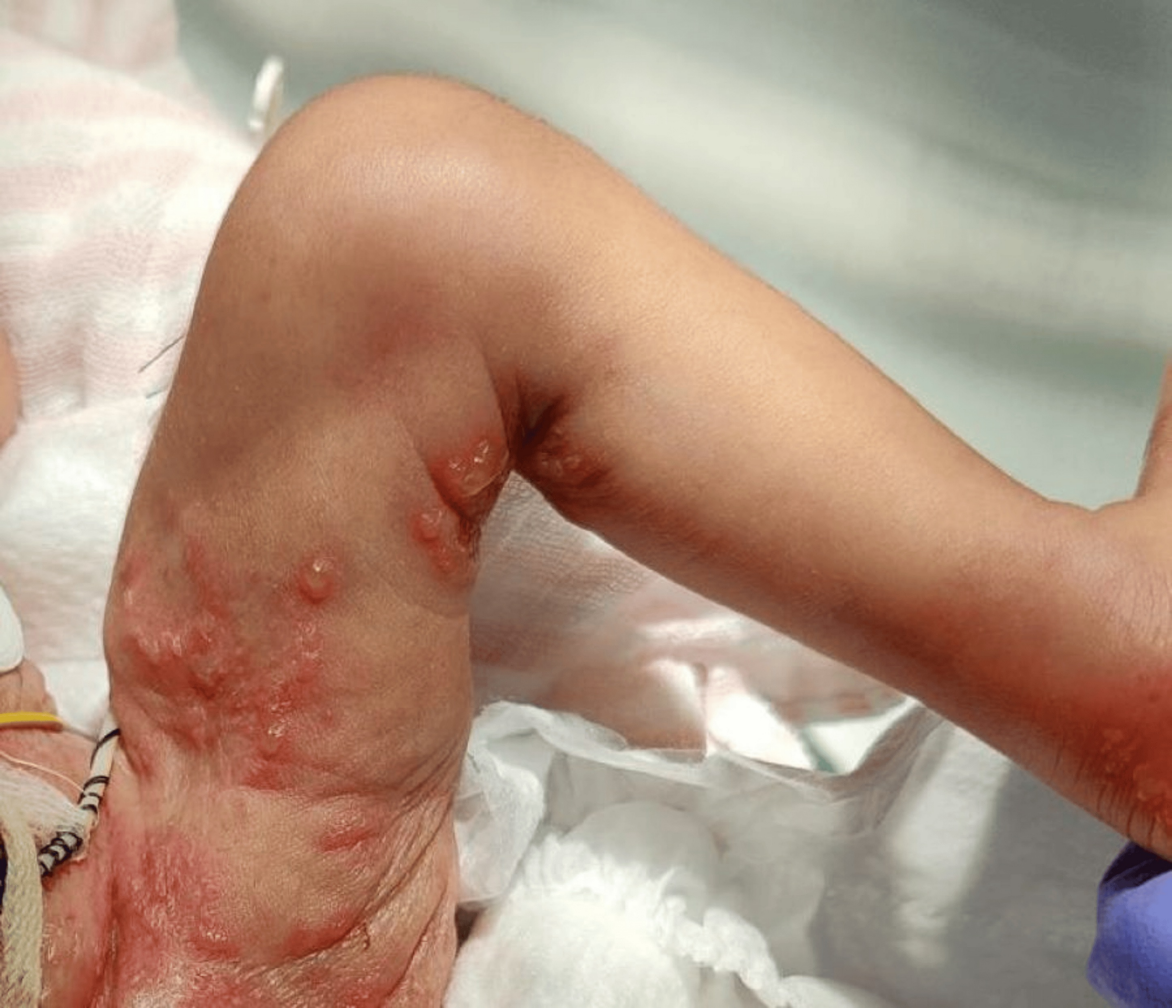
Vesicular lesions in a herpetiform pattern on the left lower limb

On the fourth day of life, the neonate developed multiple disseminated clustered vesicular lesions in a herpetiform pattern (Figure 4).

Suspecting HSV infection, intravenous acyclovir (20 mg/kg every 8 hours) was started. Skin swabs tested positive for HSV-2 by PCR (96,611,793 copies/mL). Serum testing revealed positive HSV-1 and HSV-2 IgG and negative HSV-1 and HSV-2 IgM. On that same day, the neonate exhibited clinical and laboratory deterioration, including cardiorespiratory instability and a rise in CRP to 16 mg/L. Antibiotic therapy was escalated to vancomycin (10 mg/kg every 12 hours) and cefotaxime (50 mg/kg every 12 hours). Despite maximal respiratory support, the neonate experienced recurrent episodes of apnea, profound desaturation, and bradycardia, and succumbed to her condition on the fifth day of life. Postmortem examination confirmed congenital HSV infection through microscopic examination analysis and immunohistochemical staining. Placental pathology revealed obliterative thrombotic fetal vasculopathy.

## Discussion

HSV is one of the most prevalent sexually transmitted infections. While HSV-1 is traditionally associated with herpes labialis and HSV-2 with genital herpes, the incidence of genital HSV-1 has increased, usually less severe than HSV-2, likely reflecting evolving sexual behaviors [2,5]. As in nonpregnant women, approximately two-thirds of genital HSV infections during pregnancy are asymptomatic or clinically undetectable, posing a significant diagnostic challenge [2]. This was exemplified in the present case, where the mother displayed no clinical signs of genital HSV infection before or during pregnancy. Therefore, in cases of PROM, particularly when associated with fetal malformations, HSV infection should be considered in the differential diagnosis. Prompt investigation and initiation of acyclovir therapy during pregnancy may mitigate the risk of progression to CNS involvement, potentially improving neonatal outcomes [5,6]. However, in this particular case, the severity of the CNS and systemic lesions at the time of presentation suggests that antiviral therapy, even if initiated earlier, may not have altered the final outcome.

Maternal HSV status was not assessed through virological testing. The neonate’s positive serum HSV-1 and HSV-2 IgG at birth, likely acquired transplacentally, alongside the positive HSV-2 PCR from skin swabs, suggests that the maternal HSV infection during pregnancy may have been a nonprimary first episode-a primary HSV-2 infection in a woman with pre-existing HSV-1 IgG antibodies. Nonetheless, recurrent maternal HSV-2 infection cannot be excluded.

The differential diagnosis in this case includes other congenital viral infections, such as varicella-zoster virus, CMV, and coxsackievirus. Bacterial infections, including staphylococcal species, bullous lesions associated with group B streptococcus sepsis, and hemorrhagic ulcerative lesions seen in congenital syphilis, should also be considered [4].

## Conclusions

Intrauterine HSV infection represents the rarest and most severe form of neonatal HSV infection. It is associated with a range of serious and potentially life-threatening outcomes, including spontaneous abortion, preterm birth, stillbirth, or neonatal death. Diagnosis is particularly challenging given the asymptomatic nature of most maternal HSV infections during pregnancy. As a result, intrauterine HSV infection often goes undetected until complications arise. This case highlights the importance of considering intrauterine HSV infection in the differential diagnosis of complicated pregnancies presenting with fetal malformations, as early identification and intervention could be crucial in improving maternal and neonatal outcomes.
